# Depression and Anxiety among Migrant Older Adults during the COVID-19 Pandemic in China: Network Analysis of Continuous Cross-Sectional Data

**DOI:** 10.3390/healthcare12181802

**Published:** 2024-09-10

**Authors:** Chi Zhang, Yuefan Zhao, Lei Wei, Qian Tang, Ruyue Deng, Shiyuan Yan, Jun Yao

**Affiliations:** 1 School of Health Policy and Management, Nanjing Medical University, Nanjing 211166, China; zchi@stu.njmu.edu.cn (C.Z.); zl815618@stu.njmu.edu.cn (Y.Z.); weilei@stu.njmu.edu.cn (L.W.); ruydeng@stu.njmu.edu.cn (R.D.); huangjie@stu.njmu.edu.cn (S.Y.); 2 School of Nursing, Nanjing Medical University, Nanjing 211166, China; tqlucky@stu.njmu.edu.cn; 3 Jiangsu Provincial Institute of Health, Nanjing Medical University, Nanjing 211166, China

**Keywords:** depression, anxiety, Chinese migrant older adults, network analysis, COVID-19

## Abstract

Many Chinese migrant older adults are more prone to mental health problems due to their “migrant” status. During the COVID-19 pandemic, restrictions on their mobility exacerbated these conditions. Mental health is a crucial dimension of healthy aging. Network analysis offers a novel method for exploring interactions between mental health problems at the symptom level. This study employs network analysis to examine the interactions between comorbid depressive and anxiety symptoms across different stages of the COVID-19 pandemic. Surveys were conducted from September 2019 to January 2020 (T1), September 2020 to January 2021 (T2), and September 2021 onwards (T3). Depression and anxiety symptoms were measured by the Patient Health Questionnaire-9 (PHQ-9) and the Hospital Anxiety and Depression Scale-Anxiety (HADS-A). Expected Influence (EI) and Bridge Expected Influence (Bridge EI) were used to identify central and bridge symptoms in the network. Network stability and accuracy tests were performed. Among the Chinese migrant older adults, the anxiety prevalence was 18.50% at T1, 21.11% at T2, and 9.38% at T3. The prevalence of depression was 26.95% at T1, 55.44% at T2, and 60.24% at T3. The primary central symptoms included ‘Afraid something will happen’ (A2), ‘Irritability’ (A6), ‘Panic’ (A7), ‘Feeling of worthlessness’ (D6), ‘Anhedonia’ (D1), and ‘Feeling of fear’ (A5). The major bridge symptoms included ‘Feeling of fear’ (A5), ‘Panic’ (A7), ‘Irritability’ (A6), ‘Fatigue’ (D4), ‘Anhedonia’ (D1), and ‘Depressed or sad mood’ (D2). Differences in network structure were observed across the periods. The network analysis further revealed the evolving relationships between central and bridge symptoms over time, highlighting the importance of targeted intervention strategies for central and bridge symptoms of comorbid depression and anxiety at different periods.

## 1. Introduction

COVID-19 has had a profound impact on China since it was first reported in late December 2019. Chinese people have spent three far-reaching years in the midst of the epidemic. In the context of aging in China [1], the impact of COVID-19 on the older Chinese population is enormous. The infection rate of COVID-19 in older adults is 25.3% [2], and the case fatality rate increases with age [3]. Studies have demonstrated that the high infection and case fatality rates of COVID-19 led to fear and anxiety among older adults [4]. Since the COVID-19 epidemic, elderly individuals have been considered a high-risk group, increasing the forced isolation of elderly individuals [5]. Additionally, the existence of age discrimination in society [6] has also increased the loneliness of older people [7]. Loneliness is a key predictor of depression and anxiety [8]. Several studies have illustrated the COVID-19 increased risk of depression and anxiety in different populations [9,10], as well as comorbid depression and anxiety [11]. Comorbidity of depression and anxiety produces more severe negative health outcomes than depression or anxiety alone [11]. Examples include heightened illness severity, suboptimal treatment outcomes, increased health system costs, and higher rates of suicide.

At the same time, as urbanization in China accelerates, a large number of older adults relocate from their birthplace to another city and become migrant older adults [12]. The Chinese migrant older adults refer to the population who reside outside the city of their household registration for a long time. Reasons for this phenomenon include employment, caring for children or grandchildren, and old age. According to the China Migrant Population Development Report (2018), the Chinese migrant older population is 13.5 million. They face the economic burden of work [13], language barriers [14], and changes in living conditions [15], leading to a more severe psychological burden [16]. Previous studies have indicated that migrants are more likely to suffer psychological disorders than non-migrants [17,18,19]. The sudden onset of COVID-19 has prevented Chinese migrant older adults from returning to their ancestral homes. Thus, in the context of the COVID-19 pandemic, Chinese migrant older adults face multiple stressors, which increases their anxiety and depressive symptoms.

Unlike traditional research approaches, network analysis can quantify the relationships between depressive and anxiety symptoms [20,21,22]. This is an important pathway for further understanding anxiety and depression. Multiple studies have confirmed that network analysis is useful for anxiety and depression [23,24,25]. In the theory of network analysis, anxiety and depression are viewed as a system of interacting symptoms. Networks consist of nodes (representing symptoms) and edges (representing relationships between symptoms) [26]. It provides centrality and predictability indicators for each symptom, examining its significance in the network [27]. The central symptom [20] is most strongly associated with other symptoms and may activate them. It plays a major role in the development or maintenance of the disorder. Preventive and intervention measures targeting central symptoms may be more effective [28]. In addition, when a symptom may increase the risk of developing other disorders, leading to comorbidity, this symptom is considered a bridge symptom [29,30]. Therefore, clinicians can prevent and treat comorbidities from the perspective of bridge symptoms [28,29,30]. In brief, an accurate description of these interacting symptoms is essential to explain disease mechanisms and develop targeted intervention strategies [27,31,32].

Researchers have found that the network characteristics of depression and anxiety differ between samples, such as Chinese female student nurses [23], Spanish adolescents [24], and adolescents and older adults during the lockdown of the COVID-19 pandemic [33,34]. In these studies, the symptoms “Fatigue”, “Feelings of worthlessness”, and “Irritability” have been identified as central symptoms, while “Feeling lonely” and “Feeling unloved” have been associated with an increased risk of depression and anxiety comorbidity. Older adults [35] with diabetes [36], hypertension [37], multiple chronic conditions [38], and disabilities [39] face stressors as older migrants. Their networks of anxiety and depression are cause for concern. There are differences in the networks due to the specific stressors of these groups. “Sleep difficulties” are a central symptom only among elderly males during the epidemic [35]. “Feeling of worthlessness “ and “Feeling of fear “ are central symptoms among the elderly with multiple chronic conditions [38]. “Worry too much” was the main bridge symptom for older people during COVID-19 [35], and was not manifested in other groups of older people. However, common characteristics also exist. “Worry too much” was identified as a central symptom of hypertension [37], diabetes [36], and general older adults during the COVID-19 pandemic [35]. The bridge symptom of these groups is characterized by the symptom of “Nervousness”. It also shows up as a central symptom among the elderly in diabetes [36] or COVID-19 [35]. These network analyses have provided new perspectives on the relationship and comorbidity of depression and anxiety symptoms.

However, these studies examining symptoms of anxiety and depression were limited to cross-sectional data. Network analysis using multiple continuous cross-sectional data can reveal the temporal differences in central bridge symptoms between the two symptom groups. Therefore, this study examined the anxiety and depression symptom networks of migrant older adults in Nanjing, Jiangsu Province, using three surveys from September 2019 to January 2020, September 2020 to January 2021, and September 2021. Specifically, we examined the structure of a continuous network of anxiety and depression and analyzed the most influential central symptoms and bride symptoms in the community. The aim is to elucidate differences in anxiety and depression symptom networks and identify potential targets for prevention and intervention among Chinese migrant older adults during different epidemics of COVID-19. It is hoped that targeted prevention and intervention strategies will be developed for future migrant older adults facing other stressful events.

## 2. Materials and Methods

### 2.1. Participants and Procedure

The data for this study are derived from the “Follow-up Study of Intergenerational Relations on the Mental Health Mechanism of Old Migrants”, which is funded by the China Social Science Foundation, including the data from Phase I, Phase II, and Phase III. This project was conducted from September 2019 to January 2020, September 2020 to January 2021, and September 2021 in Nanjing, China. Due to the need for a representative sample, the multilevel random sampling method was used for this research. In this project, 7 districts were first randomly selected in Nanjing (Qinhuai, Qixia, Gulou, Xuanwu, Jianye, Yuhuatai, and Jiangning District), and then 3 communities were randomly selected in each district. Migrant older adults who met the inclusion criteria in these 21 communities were enrolled. All participants were informed of the purpose of the study and consented to participate as volunteers. The necessary permission to conduct this study was obtained from the ethics committee of the university.

All participants were interviewed face-to-face using a structured questionnaire. All interviewers who had a medical research background received standardized training prior to the project. Inclusion criteria comprised adults (1) aged 50 and above, (2) having a household registration (Hukou) out of Nanjing, and (3) having lived in Nanjing ≤10 years. Exclusion Criteria were (1) elderly individuals without children or with no living children and (2) paralyzed bedridden people and people with consciousness or language communication disorders.

The survey was interrupted midway through Phase I due to a COVID-19 outbreak. The complete survey was conducted in Phase II and Phase III. Consequently, the exact sample sizes for each cross-section are different according to the inclusion and exclusion criteria: T1 = 256, T2 = 469, and T3 = 405.

### 2.2. Measures

The anxiety symptoms were measured by the Chinese version of the Hospital Anxiety and Depression Scale—Anxiety (HADS-A) [40], which comprises 7 items, each scored from “0” (not at all) to “3” (nearly every day). The sum of scores ranges from 0 to 21. Higher HADS-A scores indicate more severe anxiety symptoms. We define indicators as follows: a total score from 0 to 7 reflects no anxiety symptoms, from 8 to 10 indicates suspicious anxiety symptoms, and from 11 to 21 indicates anxiety symptoms. The internal consistency of the HADS-A in this study was excellent (Cronbach’s α = 0.744).

The depressive symptoms were measured using the Chinese version of the nine-item Patient Health Questionnaire (PHQ-9) [41]. Each item was scored from “0” (not at all) to “3” (nearly every day). The sum of scores ranges from 0 to 27. Higher total PHQ-9 scores indicate more severe depressive symptoms. The scores of 5, 10, 15, and 20 reflect mild, moderate, moderately severe, and severe depressive symptoms. The PHQ-9 has been well-validated in Chinese populations [42,43]. The internal consistency of the PHQ-9 in this study was excellent (α = 0.806).

In this study, a HADS-A total score of ≥7 was considered “having anxiety”, a PHQ-9 total score of ≥5 was considered “having depression”, and those with both a HADS-A total score of ≥7 and a PHQ-9 total score of ≥5 were considered as “having comorbid depression and anxiety”.

### 2.3. Data Analysis

#### 2.3.1. Network Estimation

The comorbidity of depression and anxiety network analyses were conducted by R software (R version 4.2.1), and statistical analyses were performed by the graph, networktools, ggplot2, and Bootnet packages [44]. In the current study, network analysis was used to examine anxiety–depression networks. Multicore correlations between all items of the PHQ-9 and HADS-A were computed based on the Graphical Gaussian Model (GGM). The Least Absolute Shrinkage and Selection Operator (LASSO) and the Extended Bayesian Information Criterion (EBIC) are models in the R package “qgraph” [22,45]. The LASSO is used to regularize the partial correlations in the represented network [46]. The EBIC hyper parameter γ was set to 0.5 to balance sensitivity and specificity [22]. The layout of the presented networks was based on the Fruchterman–Reingold algorithm [47].

The visualization of network analysis presents nodes with strong connection strength and a high number of connections near the center of the network. In the network, green edges represent positive correlations, and red edges represent negative correlations. Thicker edges mean a stronger correlation between two neighboring nodes. The network was constructed and visualized using the R package “qgraph” [45]. The predictability of each node was estimated by using the R package “MGM” [48] and represents the variance of the nodes explained by all other nodes in the network. We chose Expected Influence (EI) to characterize the central symptoms in the network [20]. This metric is more suitable for networks with positive and negative edges than the traditional central metric [49].

In this study, two disease symptom groups were predefined: the anxiety symptom group and the depressive symptom group. We use the R package “networktools” to estimate the Bridge Expected Influence (Bridge EI). Bridge EI is more appropriate than other bridge centrality for identifying bridge nodes in networks with positive and negative edges [30]. The Bridge EI of a node is the sum of the edge weights to all other symptom nodes, which reflects the importance of a single symptom connecting two symptom clusters [29,30]. The higher the expected impact value of the bridge, the higher the likelihood of activating the opposite community [30], which means that if the Bridge EI is higher, the symptoms that could prevent activation from spreading from one disease to another if deactivated are higher.

#### 2.3.2. Network Stability

The stability and accuracy of the network were examined by conducting the R package “bootnet” [22]. First, we evaluated the accuracy of edge weights by computing 95% confidence intervals using a nonparametric bootstrap (2000 bootstrap samples). Then, we evaluated the stability of node EI and node Bridge EI with a case-dropping bootstrap by calculating the correlation stability coefficient (CS-coefficient). The ideal CS-coefficient should be above 0.5 and should not be below 0.25 [22]. In addition, we conducted bootstrapped difference tests (2000 bootstrap samples and α = 0.05) for edge weights, node EI, and node bridge EI to examine whether they are significantly different from each other.

## 3. Results

### 3.1. Descriptive Statistics and Prevalence

#### 3.1.1. Descriptive Statistics

The mean age of the included participants was 61.70 ± 5.78 years (mean ± standard deviation, N = 256), of whom 71 (27.7%) were male and 185 (72.3%) were women at T1; 65.48 ± 4.56 years (mean ± standard deviation, N = 469), of whom 149 (31.8%) were male and 320 (68.2%) were women at T2, and 65.05 ± 4.59 years (mean ± standard deviation, N = 405), of whom 109 (26.9%) were male and 296 (73.1%) were women at T3. Most of the migrant older adults in the three periods were married and with a spouse and had no religious beliefs. They also considered themselves to be in fair health. More demographic variables of the participants are shown in Table 1.

#### 3.1.2. Prevalence of Anxiety and Depression

The prevalence of anxiety and depression among migrant older adults before the COVID-19 pandemic (T1) was 18.50% and 26.95%, respectively. The prevalence of anxiety and depression among migrant older adults in the strict control period after the COVID-19 pandemic (T2) was 21.11% and 55.44%, respectively. The prevalence of anxiety and depression among migrant older adults in the normative management following the COVID-19 pandemic (T3) was 9.38% and 60.24%, respectively. Table 2 shows the prevalence of each level of anxiety and depression.

Table 3 shows the abbreviations, mean scores, and standard deviations for each variable in the current network.

**Figure 1:**
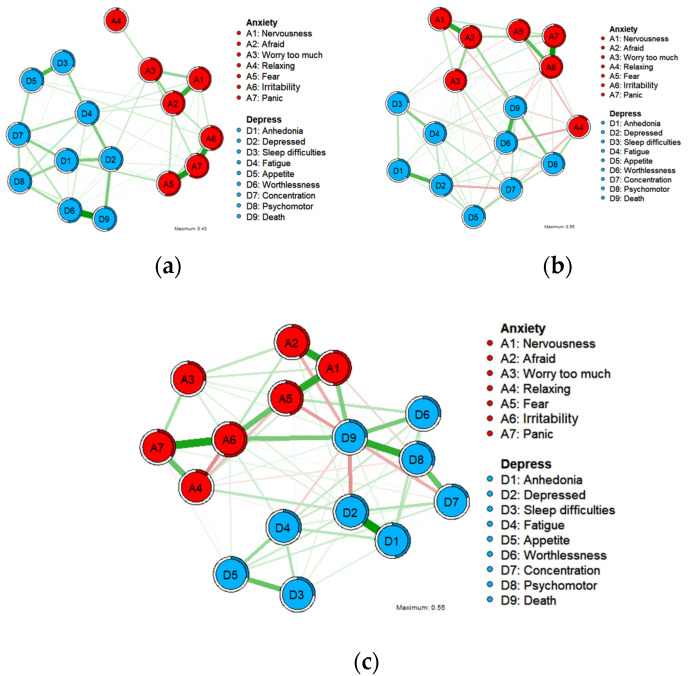
Undirected networks of anxiety and depression symptoms at T1 ((**a**); N = 256), at T2 ((**b**); N = 469), and at T3 ((**c**); N = 405). Notes: A1 = Nervousness or anxiety. A2 = Afraid something will happen. A3 = Worry too much. A4 = Trouble relaxing. A5 = Feeling of fear. A6 = Irritability. A7 = Panic. D1 = Anhedonia. D2 = Depressed or sad mood. D3 = Sleep difficulties. D4 = Fatigue. D5 = Appetite changes. D6 = Feeling of worthlessness. D7 = Concentration difficulties. D8 = Psychomotor agitation/retardation. D9 = Thoughts of death. Green edges indicate positive associations between nodes, while red edges indicate negative associations.

**Figure 2:**
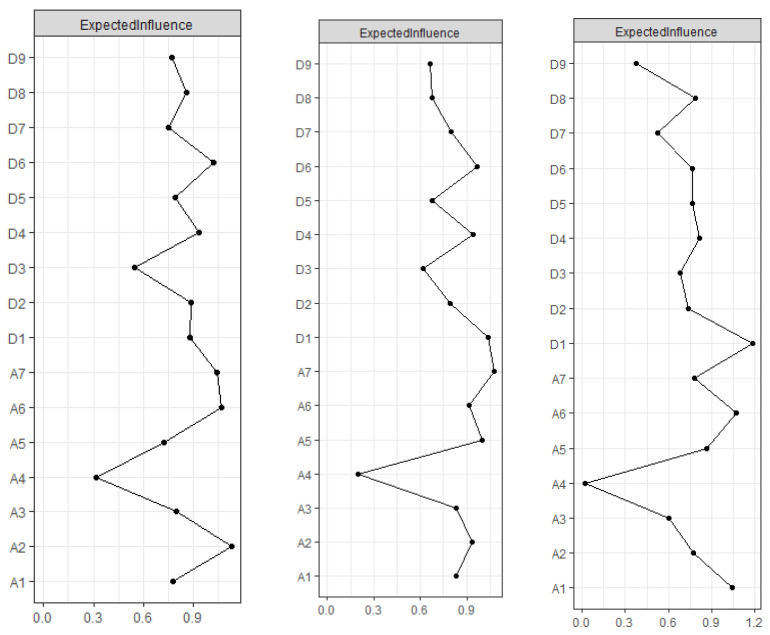
Symptom centrality plots based on the Expected Influence (EI) centrality index of cross-sectional undirected networks (T1, T2, T3) and longitudinal change trajectory slope network. Notes: A1 = Nervousness or anxiety. A2 = Afraid something will happen. A3 = Worry too much. A4 = Trouble relaxing. A5 = Feeling of fear. A6 = Irritability. A7 = Panic. D1 = Anhedonia. D2 = Depressed or sad mood. D3 = Sleep difficulties. D4 = Fatigue. D5 = Appetite changes. D6 = Feeling of worthlessness. D7 = Concentration difficulties. D8 = Psychomotor agitation/retardation. D9 = Thoughts of death.

**Figure 3:**
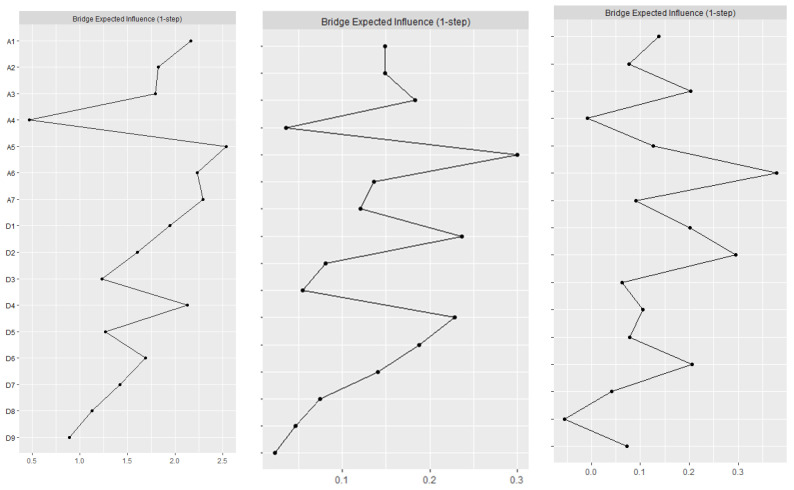
Symptom bridge centrality plots based on the Bridge Expected Influence (Bridge EI) index of cross-sectional undirected networks (T1, T2, T3) and longitudinal change trajectory slope network. Notes: A1 = Nervousness or anxiety. A2 = Afraid something will happen. A3 = Worry too much. A4 = Trouble relaxing. A5 = Feeling of fear. A6 = Irritability. A7 = Panic. D1 = Anhedonia. D2 = Depressed or sad mood. D3 = Sleep difficulties. D4 = Fatigue. D5 = Appetite changes. D6 = Feeling of worthlessness. D7 = Concentration difficulties. D8 = Psychomotor agitation/retardation. D9 = Thoughts of death.

Figure 1 depicts the three undirected networks for anxiety and depression. The centrality and bridge centrality rankings of each node are shown in Figure 2 and Figure 3.

### 3.2. Network Structure

#### 3.2.1. Stage of Symptoms

Before the COVID-19 pandemic (T1), out of 120 possible edges, 69 edges were nonzero (64.17%), and all edges were positively correlated. The predictability of each symptom is displayed in the form of a circular pie chart. The mean predictability is 0.42, indicating that an average of 42% of the variance of each node can be explained by adjacent nodes. The anxiety symptom ‘Panic’ (A7) had the highest predictability, indicating that 61% of the variance could be explained by neighbors.

During the strict control period after the COVID-19 pandemic (T2), out of the 120 possible edges, 78 were nonzero (65%), with a total of 7 negatively correlated edges, with the rest of the sides being positive. The predictability of each symptom is shown in the form of a circular pie chart. The mean predictability is 0.40, indicating that an average of 40% of the variance of each node can be explained by the neighboring nodes. The anxiety symptom ‘Irritability’ (A6) had the highest predictability, indicating that 67% of its variance could be explained by neighbors.

In the normative management following the COVID-19 pandemic (T3), out of the 120 possible edges, 78 were nonzero (65%), with a total of 7 negatively correlated edges. The rest of the edges are positive. The predictability of each symptom is shown in the form of a circular pie chart. The mean predictability is 0.33, indicating that an average of 33% of the variance of each node can be explained by adjacent nodes. The anxiety symptom ‘Irritability’ (A6) had the highest predictability, indicating that 55% of its variance could be explained by neighbors.

#### 3.2.2. Central Symptoms

In the centrality index Expected Influence (EI), before the COVID-19 pandemic (T1), ‘Afraid something will happen’ (A2) has the highest EI, followed by nodes ‘Irritability’ (A6), ‘Panic’ (A7), and ‘Feeling of worthlessness’ (D6), suggesting that these four symptoms are significant and influential in the structure of this period. The correlation stability coefficient (CS-coefficient) of EI was 0.438, indicating that the estimates of the nodal expectancy effect were sufficiently stable. During the strict control period after the COVID-19 pandemic (T2), ‘Panic’ (A7) had the highest EI, followed by nodes ‘Anhedonia’ (D1), ‘Feeling of fear’ (A5), and ‘Feeling of worthlessness’ (D6), suggesting that these four symptoms are important and influential in understanding the structure of depression and anxiety of migrant older adults during this period. The CS-coefficient of EI was 0.672, indicating that the estimation of the nodal expectation impact was sufficiently stable. In the normative management following the COVID-19 pandemic (T3), ‘Anhedonia’ (D1) had the highest EI, followed by ‘Irritability’ (A6) and ‘Nervousness or anxiety’ (A1), indicating that these are central symptoms during T3. The CS-coefficient of EI was 0.516, indicating that the estimation of nodal expectation influence was sufficiently stable.

#### 3.2.3. Bridge Symptoms

For bridge Expected Influence (Bridge EI), before the COVID-19 pandemic (T1), ‘Feeling of fear’ (A5), ‘Panic’ (A7), ‘Irritability’ (A6), and ‘Fatigue’ (D4) were the most critical bridge symptoms that connected the depression and anxiety communities. This suggests that in the current network, these four symptoms have the strongest ability to increase the risk of depression to anxiety transmission. The correlation stability coefficient (CS-coefficient) of the Bridge EI was 0.285, indicating that the estimated expected impact of nodal bridges is sufficiently stable. The strict control period after the COVID-19 pandemic (T2), ‘Feeling of fear’ (A5), ‘Anhedonia’ (D1), and ‘Fatigue’ (D4) were the most critical bridge syndromes connecting depression and anxiety communities. This suggests that ‘Feeling of fear’ (A5) has the strongest ability to increase the risk of transmission of anxiety to depression. ‘Anhedonia’ (D1) and ‘Fatigue’ (D4) have the strongest ability to increase the risk of transmission to anxiety in the current network. The CS-coefficient of Bridge EI was 0.516, indicating that the estimates of the expected impact of nodal bridges are sufficiently stable. In the normative management following the COVID-19 pandemic (T3), ‘Irritability’ (A6) and ‘Depressed or sad mood’ (D2) are the most critical bridge syndromes connecting the depressed and anxious communities. This suggests that in the current network, ‘Irritability’ (A6) has the strongest ability to increase the risk of transmission from anxiety to depression, and ‘Depressed or sad mood’ (D2) has the strongest ability to increase the risk of transmission to anxiety. The CS-coefficient of Bridge EI was 0.44, indicating that the estimated expected impact of nodal bridging is sufficiently stable.

## 4. Discussion

To our knowledge, this is the first study to describe the network structure of depression and anxiety comorbidity and its development among migrant older adults. In addition to the novel study population, a key strength of this research is its multiregional data collection across three phases. Fortunately, the first data were collected before the onset of COVID-19. This allows for the assessment of causal relationships associated with symptom changes over time, making it the first and only study in China. In this study, a network analysis was employed to model the relationship between anxiety and depressive symptoms in a sample of migrant older adults in Nanjing at three time points before and after the COVID-19 outbreak. It provided an opportunity to gain insight into how anxiety and depressive symptoms in migrant older adults developed and were linked in this unprecedented historical context.

Initially, a Gaussian graphical model with LASSO penalty regularization was employed to identify the network structure of anxiety and depression. Using this network, the strongest edges for each psychiatric disorder were identified, which is consistent with previous network studies examining the comorbidity of depressive and anxiety symptoms. The connection between anxiety and depressive symptoms appeared to evolve over time. A high edge strength and a link between ‘Feeling of fear’ (A5) and ‘Panic’ (A7) were noted before the onset of the COVID-19 epidemic (T1). The link between these two symptoms is consistent with studies of public hospital physicians [25] but diminished after the onset of the COVID-19 epidemic (T2 and T3). A similar typological phenomenon also occurred between ‘Feeling of worthlessness’ (D6) and ‘Thoughts of death’ (D9). In contrast, the association between ‘Anhedonia’ (D1) and ‘Depressed or sad mood’ (D2) strengthened with the onset of the epidemic. The edge strength of these two symptoms was normal before the COVID-19 epidemic (T1) but became the highest edge strength after the onset of the epidemic (T2 and T3). This phenomenon was consistent with findings in samples from medical staff [50], Macau residents [34], and the elderly population [51] during the COVID-19 epidemic but was not demonstrated before COVID-19 [34]. Further validation may be required to confirm that the strong edges of ‘Anhedonia’ (D1) and ‘Depressed or sad mood’ (D2) are specific to particular samples in the context of the COVID-19 pandemic.

This study also examined the most central nodes and the most influential bridge nodes. Throughout the phases, the most central anxiety symptom was ‘Irritability’ (A6), and the most central depressive symptom was ‘Anhedonia’ (D1). It indicated that these symptoms play the most critical role in maintaining the overall symptom network. According to previous studies, irritability has been defined as a low threshold for experiencing anger in response to frustration [52]. Migrant older adults are highly vulnerable to anger due to their inherent instability and the inconvenience of experiencing epidemic prevention and control. At the same time, older adults are more likely to be anhedonia and have less curiosity than younger adults [51]. Anhedonia is not reflected in the networks of the elderly with diabetes, hypertension, disability, and multiple chronic diseases, but it is prominent in Chinese migrant older adults. This highlights that “mobility” significantly influences the source of pleasure. In a status of anhedonia, the internal attribution of the elderly is helpless and the root of the source of depression or sadness. It is a reactive chain [51]. It can be hypothesized that psychological interventions aimed at calming migrant older adults and stimulating interest in life, as well as physical activity interventions [53,54], may be effective in improving symptoms to alleviate the potential of other symptoms in the anxiety–depression symptom network.

‘Nervousness or anxiety’ (A1) appeared as a central symptom in different studies but was not evident in the migrant older adult before the COVID-19 pandemic (T1). It gradually emerged as a central symptom after the onset of COVID-19 (T2 and T3). This phenomenon has been observed in samples of older adults [51] after the COVID-19 epidemic. Nervousness is characterized by a feeling of restlessness in response to an impending threat. The culture or context of different countries and regions can explain the symptoms [55]. In traditional Chinese families, older adults are particularly concerned about their family’s difficulties, usually due to their cultural characteristics [56], such as illness, lack of income, or being perceived as a burden to their children due to physical illness and low socioeconomic status [57]. Furthermore, migrant older adults, in addition to the worries and stresses caused by these cultural backgrounds, the economic burden of work [13], language barriers [14], changes in living conditions [15], and regional discrimination further add to the psychological burden of migrant older adults. Similarly, ‘Anhedonia’ (D1), which was a less prominent central symptom before the COVID-19 pandemic (T1), became the most central depressive symptom after the onset of COVID-19 (T2 and T3). Furthermore, ‘Afraid something will happen’ (A2) and ‘Depressed or sad mood’ (D2) were the central symptoms in migrant older adults before the COVID-19 pandemic (T1) and psychiatric patients and Philippine housemates [20,58]. The centralities of ‘Afraid something will happen’ (A2) and ‘Depressed or sad mood’ (D2) gradually diminished with the continuation of the sequestration time after the onset of COVID-19 and were no longer central symptoms (T2 and T3).

The variation in central symptoms of anxiety and depression over time may be associated with gradual changes in perceived changes in mental stress among immigrant older adults over three periods. So, the interesting thing is that the perception of the COVID-19 threat may decrease at T3. ‘Panic’ (A7) and ‘Feeling of worthlessness’ (D6) are the central symptoms before and after the COVID-19 outbreak (T1 and T2), but with normalized management of the COVID-19 epidemic (T3) and the older adults themselves trying to return to normal, the perception of the COVID-19 threat may decrease, while making panic and self-denial gradually decrease [59].

In spite of the central symptoms, we also investigated the most influential bridge nodes [30,33] between anxiety and depression at different periods, including ‘Nervousness or anxiety’ (A1), ‘Feeling of fear’ (A5), ‘Panic’ (A7), ‘Anhedonia’ (D1), ‘Depressed or sad mood’ (D2), ‘Fatigue’ (D4), and ‘Feeling of worthlessness’ (D6). First of all, ‘Anhedonia’ (D1), ‘Feeling of worthlessness’ (D6), and ‘Worry too much’ (A3) remained the characteristics of bridge symptoms in the anxiety and depression networks for a long time. In other words, these symptoms served as the bridge between anxiety and depression. However, because GGM networks are undirected, they cannot identify whether a symptom is the cause of another symptom and vice versa. First, ‘Feeling of fear’ (A5) and ‘Fatigue’ (D4) were the predominant bridge symptoms at T1 and T2, as was found in the study of Macau residents [34] after the COVID-19 outbreak. However, with the normalization of COVID-19 management (T3), these symptoms gradually ceased to serve as bridge symptoms. Second, ‘Depressed or sad mood’ (D2) became a bridge symptom at T2, which is different from the study conducted in Harbin, China, during the COVID-19 pandemic. The discrepancy may be due to the fact that the study in Harbin was conducted on older people following a strict lockdown in 2021, while our study was conducted on migrant older adults during the period of easing restrictions as the epidemic subsided. Under regular management of COVID-19 (T3), ‘Depressed or sad mood’ (D2) became the main bridge symptom, which is consistent with the findings of a study conducted in the same period in China among clinicians in public hospitals [25]. It was also a bridge symptom among Filipino domestic workers during the COVID-19 pandemic [58]. In network theory [30], psychiatric comorbidity can be reduced if the bridge symptoms that connect comorbidity syndromes/diseases can be improved. Thus, interventions that target these bridge symptoms may reduce the risk of concurrent depression and anxiety among migrant older adults.

Specifically, this study compared changes in anxiety and depression networks over time over different periods. Previous studies have relied primarily on cross-sectional data [33,60], with only a few exceptions [59,61,62]. The findings of our study suggest that the longitudinal network provides supplementary insights beyond merely replicating the cross-sectional network. In addition, we investigated central symptoms and influential bridge symptoms between anxiety and depression. Our results may inform the development of personalized prevention and intervention strategies that account for symptom heterogeneity. An increasing number of studies are now applying network analysis to clinical practice [63]. Clinicians can achieve breakthroughs in the treatment of physical illnesses by focusing on mental health issues [64], which network analysis can facilitate. Finally, the findings of this study may enhance the understanding and management of anxiety and depression symptoms and support the recovery of psychological well-being in older adults after the liberalization of COVID-19 restrictions in China.

However, our study also has some limitations. The reliance on self-reports rather than clinical interviews may limit the reliability of our findings. Due to the sudden onset of the COVID-19 epidemic, the data collected before the COVID-19 pandemic (T1) were insufficient. However, additional participants were recruited later to supplement the data. There was still an insufficient amount of T1 data. Additionally, the participants were, on average, migrant older adults (>50 years old) in Nanjing, which may limit the applicability of the results to other age groups. Furthermore, the more developed economic and cultural level of the city may affect the generalizability of the findings to a national level.

## 5. Conclusions

This study used network analysis to describe the structural pathways of anxiety and depression over multiple periods. The strength of the edges between ‘Feeling of fear’ (A5) and ‘Panic’ (A7) decreased over time. In contrast, an increased edge strength was observed between ‘Anhedonia’ (D1) and ‘Depressed or sad mood’ (D2). The most central symptoms of anxiety and depression were found to be ‘Irritability’ (A6) and ‘Anhedonia’ (D1). Simultaneously, ‘Nervousness or anxiety’ (A1) and ‘Anhedonia’ (D1) gradually became central symptoms following the onset of COVID-19. The bridge symptoms between anxiety and depression include ‘Nervousness or anxiety’ (A1), ‘Feeling of fear’ (A5), ‘Panic’ (A7), ‘Anhedonia’ (D1), ‘Depressed or sad mood’ (D2), ‘Fatigue’ (D4), and ‘Feeling of worthlessness’ (D6). These symptoms play a role at different times. These findings illustrate how anxiety and depression can lead to the development and maintenance of comorbidity over time.

## Figures and Tables

**Table 1:** Sociodemographic information of the participants Mean ± SD (range) N (%).

		T1 (N = 256)		T2 (N = 469)		T3 (N = 405)	
Variables	Category	N	Mean ± SD/ Percentage (%)	N	Mean ± SD/ Percentage (%)	N	Mean ± SD/ Percentage (%)
Age		256	61.70 ± 5.78	469	65.48 ± 4.56	405	65.05 ± 4.59
Sex	Male	71	27.7	149	31.8	109	26.9
	Female	185	72.3	320	68.2	296	73.1
Marital status	Divorced or widowed	32	12.5	71	15.1	56	13.8
	Married and with a spouse	224	87.5	398	84.9	349	86.2
Education level	Primary school or lower	98	38.3	264	56.3	165	40.8
	Junior or senior high school	135	52.7	184	39.2	207	51.1
	College or higher	23	9.0	21	4.5	33	8.1
Religious belief	NO	224	87.5	398	84.9	343	84.7
	YES	32	12.5	71	15.1	62	15.3
Household registration	Rural	150	58.6	347	74.0	251	62.0
	Town	105	41.0	122	26.0	154	38.0
Yearly income	<5000 RMB	91	35.5	173	36.9	173	42.7
	5000 RMB–10,000 RMB	28	10.9	109	23.2	36	8.9
	10,000 RMB–40,000 RMB	99	38.7	136	29.0	159	39.3
	>40,000 RMB	38	14.8	51	10.9	37	9.2
Self-reported physical health	Poor	22	8.6	23	4.9	26	6.3
	Fair	143	55.8	322	68.7	263	65.0
	Good	91	35.6	124	26.5	116	28.7

**Table 2:** The prevalence of each level of anxiety and depression.

		T1 (N = 256)	T2 (N = 469)	T3 (N = 405)
	Level of Anxiety and Depression	Prevalence		
Anxiety symptoms (HADS-A)	No anxiety symptoms	88.28%	78.89%	90.62%
	Suspected anxiety symptoms	9.38%	16.84%	7.90%
	Have anxiety symptoms	2.34%	4.26%	1.48%
Depression symptoms (PHQ-9)	No depressive symptoms	73.05%	44.56%	39.75%
	Mild depressive symptoms	20.70%	42.86%	52.84%
	Moderate depressive symptoms	5.08%	10.23%	6.67%
	Moderate to severe depressive symptoms	0.78%	2.13%	0.74%
	Severe depressive symptoms	0.39%	0.21%	0%

**Table 3:** Abbreviations, mean scores, and standard deviations for each variable.

		T1 (N = 256)			T2 (N = 469)			T3 (N = 405)		
Variables	Abbreviation	Mean	SD	Predictability	Mean	SD	Predictability	Mean	SD	Predictability
Anxiety symptoms (HADS-A)										
Nervousness or anxiety	A1	0.45	0.54	0.45	0.53	0.59	0.44	0.69	0.51	0.50
Afraid something will happen	A2	0.46	0.64	0.45	0.59	0.68	0.58	0.63	0.57	0.42
Worry too much	A3	0.34	0.54	0.43	0.54	0.65	0.42	0.42	0.56	0.27
Trouble relaxing	A4	0.92	0.90	0.05	1.04	0.95	0.14	0.74	0.80	0.11
Feeling of fear	A5	0.31	0.52	0.54	0.37	0.54	0.42	0.62	0.58	0.45
Irritability	A6	0.45	0.67	0.51	0.69	0.81	0.67	0.72	0.58	0.55
Panic	A7	0.41	0.72	0.61	0.74	0.85	0.66	0.47	0.60	0.35
Depression symptoms (PHQ-9)										
Anhedonia	D1	0.46	0.77	0.43	0.71	0.71	0.36	0.79	0.67	0.47
Depressed or sad mood	D2	0.37	0.59	0.43	0.55	0.65	0.45	0.47	0.61	0.36
Sleep difficulties	D3	0.80	0.93	0.30	1.01	0.90	0.25	1.11	0.81	0.27
Fatigue	D4	0.54	0.72	0.44	0.86	0.81	0.38	0.74	0.64	0.29
Appetite changes	D5	0.39	0.75	0.31	0.45	0.66	0.28	0.61	0.69	0.28
Feeling of worthlessness	D6	0.15	0.44	0.52	0.42	0.63	0.39	0.39	0.53	0.28
Concentration difficulties	D7	0.29	0.61	0.36	0.61	0.71	0.23	0.42	0.56	0.21
Psychomotor agitation/retardation	D8	0.21	0.50	0.37	0.62	0.66	0.31	0.52	0.59	0.31
Thoughts of death	D9	0.06	0.31	0.46	0.24	0.57	0.34	0.18	0.43	0.21

## Data Availability

On reasonable request, these data may be made available from the corresponding author.
